# Interactive CardioVascular and Thoracic Surgery—2021 reviewers

**DOI:** 10.1093/icvts/ivac092

**Published:** 2022-04-05

On behalf of EACTS and EBCP, the Editor-in-Chief wishes to thank the following colleagues who have voluntarily given their valued time and effort to reviewing papers for ICVTS in 2021.

Tomonobu Abe

Firas Abu Akar

Anders Ahlsson

Diana Aicher

Clemens Aigner

Ahmet Ruchan Akar

Payam Akhyari

Ousama Al Habash

Zohair Al Halees

Johannes Albes

Maria Inmaculada Alcalde Mayayo

Cem Alhan

Yousef Alharbi

Kamran Ali

Jason Ali

Marco Alifano

Nelson Alphonso

Bahaaldin Alsoufi

Peter Alston

Antonio Alvarez

Marcello Ambrogi

Luca Ampollini

Olga Ananiadou

Rafael Andrade

Martin Andreas

Eleni-Rosalina Andrinopoulou

Dimitrios Angouras

Phillip Antippa

Manuel Antunes

Shabana Anwar

Beatrice Aramini

Arunkumar Arasappa

Claudia Arenz

Olgun ARIBAŞ

Rakesh Arora

Sinan Arsan

Gokhan Arslan

Panagiotis Artemiou

Tohru Asai

Mitsuru Asano

Tim Attmann

John Augoustides

Erle Austin

Rüdiger Autschbach

Emile Bacha

Jean Bachet

Frank Baciewicz Jr.

Catalin Badiu

Nikolaos Baikoussis

Pietro Bajona

Farhad Bakhtiary

Husam Balkhy

Ko Bando

Toru Bando

Luciano Barbato

Mohammad Bashir

Hasan Batirel

Victor Bautista-Hernandez

Thomas Beaver

J.F. Bechtel

Erik Beckmann

Eric Bedard

Jose Belda-Sanchis

Emre Belli

Denis Berdajs

Mikolaj Berezowski

Tim Berger

Luca Bertoglio

Pietro Bertoglio

Luca Bertolaccini

Amit Bhargava

Fausto Biancari

Benjamin Bidstrup

Rocco Bilancia

Henrik Bjursten

Antonio Bobbio

Dietmar Boethig

Ad Bogers

Lyubomyr Bohuta

Servet Bölükbas

Nikolaos Bonaros

Johannes Bonatti

Stefano Bongiolatti

Andreas Böning

Michael Borger

Mario Borin

Ahmed Boseila

Petre Botianu

Tomaso Bottio

Thierry Bove

Pierre-Yves Brichon

Alessandro Brunelli

Vito Bruno

Hans Ulrich Burger

Nicholas Burris

Lucio Cagini

Ufuk Cagirici

Antonio Calafiore

Duke Cameron

Alessio Campisi

Ayten Cangir

Christopher Cao

Giovanni Carboni

Giuseppe Cardillo

Manuel Carnero-Alcazar

Matthew Carr

Angelo Carretta

Raymond Cartier

Edward Caruana

Filip Casselman

Patrizio Castelli

Javier Castillo

Thierry Caus

Alberto Cereda

Alfredo Cerillo

Stepan Cerny

Laurens Ceulemans

David Chambers

Yin-Kai Chao

Andrew Chatzis

Toyofumi Chen-Yoshikawa

Cai Cheng

Yeung-Leung Cheng

Giovanni Alfonso Chiariello

Jong Bum Choi

Torsten Christ

Michael Chu

Sertac Cicek

Paola Ciriaco

Julie Cleuziou

Andrea Colli

Miguel Congregado

Lenard Conradi

Victor Costache

Antonio D'Andrilli

Xavier D'Journo

Mario Giovanni Gerardo D'Oria

Niccolo Daddi

Mark Danton

John Dark

Gail Darling

Joao-Carlos Das-Neves-Pereira

Hiroshi Date

Hitendu Dave

Piroze Davierwala

Michele De Bonis

Laurent De Kerchove

Filip De Somer

Georges Decker

Victoria Delgado

Andrea Dell'Amore

Alessandro Della Corte

Stefanos Demertzis

Michelle Demetres

Vitaly Demyanchuk

Manan Desai

Shriprasad Deshpande

Christian Detter

Luca Di Marco

Michele Di Mauro

Giovanni Dialetto

Vanessa Diaz-Ravetllat

Ioannis Dimarakis

Torsten Doenst

Daniel-Sebastian Dohle

Dimitrios Dougenis

Daniel Duerschmied

Julia Dumfarth

Joel Dunning

Leonardo Duranti

Ahmet Durukan

Friedrich Eckstein

Frank Edwin

Stefano Elia

Shunsuke Endo

Abdullah Erdogan

Giampiero Esposito

Anthony Estrera

Pierre-Emmanuel Falcoz

Wentao Fang

Enrico Ferrari

Alfonso Fiorelli

Felix Fleissner

Thierry Folliguet

Francesco Formica

Katrien Francois

Jorge Freixinet

Stephen Fremes

Örjan Friberg

Buntaro Fujita

Ikuo Fukuda

Toshihiro Fukui

Henning Gaissert

Domenico Galetta

Lorenzo Galletti

Michele Gallo

Ovidio García-Villarreal

Gaetano Gargiulo

Cengiz Gebitekin

Sandro Gelsomino

Gino Gerosa

Olivier Ghez

Daniyar Gilmanov

Leonard Girardi

Marie-Noelle Giraud

Evaldas Girdauskas

David Glineur

Donald Glower

Walter Gomes

Alessandro Gonfiotti

Arun Gopalakrishnan

Dominique Gossot

Roman Gottardi

Martin Grabenwöger

Stuart Grant

Valentina Grazioli

William Grossi

Jürg Grünenfelder

Dominik Guensch

Francesco Guerrera

Robert Guidoin

Rui Haddad

Jeffrey Hagen

Peter Haldenwang

Henrik Hansen

Jan Hinnerk Hansen

Emma Hansson

Amer Harky

Majid Harmouche

Wael Hassanein

Tim Hayes

Guo-Wei He

David Healy

Claudia Heilmann

Paul Heinisch

Khosro Hekmat

Olli Helminen

Daniel Hernandez-Vaquero

Samuel Heuts

Hirofumi Hioki

Vibeke Hjortdal

Maurice Hogan

Tomas Holubec

Alexander Horke

Amir-Reza Hosseinpour

Po-Kuei Hsu

Hung-Lung Hsu

Cheng-Long Huang

Christoph Huber

Steven Hunter

Mohsen Ibrahim

Hajime Ichikawa

Hitoshi Igai

Yasunori Iida

Ilkka Ilonen

Moritz Immohr

Yoshito Inoue

Masayoshi Inoue

Tadashi Isomura

Krishna Iyer

Parvathi Iyer

Hironori Izutani

Samuel Jacob

Oyvind Jakobsen

Katarzyna Januszewska

Marcelo Jimenez

Federica Jiritano

Ivan Joalsen

Richard Jonas

Klaus Kaier

Larry Kaiser

David Kalfa

Klaus Kallenbach

Antonios Kallikourdis

Markus Kamler

Fikret Kanat

Yukihiro Kaneko

Daisuke Kaneyuki

Emmanouil Kapetanakis

Tevfik Kaplan

Riken Kawachi

Masafumi Kawamura

Hakki Kazaz

Gunter Kerst

Suresh Keshavamurthy

Krishna Khargi

Doosang Kim

Naoyuki Kimura

Takeshi Kinoshita

Attila Kiss

Tadashi Kitamura

Soichiro Kitamura

Patrick Klein

Christoph Knosalla

Mladen Kocica

Luca Koechlin

Markus Kofler

Alexander Kogan

Philippe Kolh

Stoyan Kondov

Igor Konstantinov

Tomislav Kopjar

Andreas Koster

Tibor Krajc

Markus Krane

George Krasopoulos

Maximilian Kreibich

Tobias Krüger

Bartosz Kubisa

Takashi Kunihara

Thomas Kuntze

Paul Kurlansky

Francois Lacour-Gayet

Tanel Laisaar

Ludwig Lampl

Stephen Large

Francoise Le Pimpec-Barthes

Cheul Lee

Eric Lehr

Miia Lehtinen

Massimo Lemma

Sergey Leontyev

Mario Lescan

Gunda Leschber

Oliver Liakopoulos

Peter Licht

Antonio Loforte

Daniel Loisance

Jose Lopez-Menendez

Roberto Lorusso

Julian Losanoff

Attilio Lotto

Gianluca Lucchese

Marco Lucchi

Giovanni Battista Luciani

Heyman Luckraz

Wei-Guo Ma

Sven Meier

Vladimir Makaloski

Tarek Malas

Supreet Marathe

Mateo Marin-Cuartas

Massimiliano Marrocco-Trischitta

Thomas Martens

Sven Martens

Andreas Martens

Sofia Martin-Suarez

Akira Marui

Stefano Mastrobuoni

Pasquale Mastroroberto

Hitoshi Matsuda

Haruhisa Matsuguma

Peter Matt

Sandro Mattioli

Katya Mauff

Giulio Maurizi

Constantine Mavroudis

Alessandro Mazzola

Alessandro Mazzucco

Massimiliano Meineri

Samuel Menahem

Philippe Menasche

Lorenzo Menicanti

Ari Mennander

Olaf Mercier

Carlos Mestres

Antonio Miceli

Guido Michielon

Stephanie Mick

Milan Milojevic

Kenji Minatoya

Martin Misfeld

Paul Modi

Thomas Modine

Thomas Molnar

Emilio Monguió

Luiz Moreira

Paula Moreno

Akimasa Morisaki

Marco Moscarelli

Michael Mueller

Ludwig Müller

Giacomo Murana

Takashi Murashita

Diego Murillo Brito

Patrick Myers

Hani Najm

Hiroyuki Nakajima

Masakazu Nakao

Francesco Nappi

Pradeep Narayan

Antonio Nenna

Eugenio Neri

Calvin Ng

Dumbor Ngaage

Lars Niclauss

Lars Nölke

Nuria Novoa

Shahab Nozohoor

Satoshi Numata

Yoshie Ochiai

Inger Oey

Hitoshi Ogino

Kenji Okada

Meinoshin Okumura

Guven Olgac

Burak Onan

Masamichi Ono

Kyohei Oyama

Nobuchika Ozaki

Kanat Ozisik

Davide Pacini

Richard Page

Leonardo Paim

George Palatianos

Philip Pang

Kay-Hyun Park

In Kyu Park

Andrew Parry

Francesco Patane

Gregory Pattakos

Syed Peer

Louis P. Perrault

Bertrand Perrin

Sven Peterss

Joachim Photiadis

Gabriele Piffaretti

Hans Pilegaard

Clarence Pingpoh

Antonios Pitsis

Jan Poelaert

Eugenio Pompeo

Alain Poncelet

Michael Poullis

Marco Pozzi

Alberto Pozzoli

René Prêtre

Ourania Preventza

Elena Prisciandaro

Sylvio Provenzano

Prakash Punjabi

Lawand Qaradaghi

Rizwan Qureshi

Mohamed Rahouma

Karthik Ramakrishnan

Ian Ramnarine

Madhuri Rao

Filippo Rapetto

Ardawan Rastan

Rajashekara Reddy

Shekar Reddy

Hermann Reichenspurner

J Reinersman

Diana Reser

Oliver Reuthebuch

Araz Rezaeikivi

Zaccaria Ricci

Isabel Rodriguez

Albert Rodríguez-Fuster

Sonia Ronchey

Marc Ruel

André Rüffer

Bartosz Rylski

Michel Pompeu Sá

Jörg Sachweh

Satoshi Saito

Alessandra Sala

Mohamed Salama

Antonio Salsano

Giuseppe Santarpino

Nishant Saran

Ulrik Sartipy

Milton Saute

Noriyoshi Sawabata

Paolo Scanagatta

Thomas Schachner

Hans-Joachim Schäfers

Michael Scharfschwerdt

Stefano Schena

David Schibilsky

Georg Schlachtenberger

Jürg Schmidli

Christoph Schmitz

Richard Schuessler

Thomas Schwann

Burkhardt Seifert

Massimo Sella

Paul Sergeant

Michael Shackcloth

Mohammad Shadmehr

Sharaf-Eldin Shehada

Norihiko Shiiya

Yukitoshi Shirakawa

Dominique Shum-Tim

Pranava Sinha

Francis Smit

Freyja Smolle-Jüttner

Yoshiharu Soga

Makoto Sonobe

Rodrigo Soto

Stefan Sponholz

Mila Stajevic

Alessia Stanzi

Alessandro Stefani

Serban Stoica

Gengo Sunagawa

Tk Susheel Kumar

Piotr Suwalski

Staffan Svenmarker

Jan Svennevig

Ben Swinkels

Federico Tacconi

Sachin Talwar

Derrick Tam

Lijie Tan

James Tatoulis

Adele Tessitore

Stefan Thelin

Alister Thomas

Pascal Thomas

Matthieu Thumerel

Kaushal Tiwari

Koichi Toda

Alper Toker

Masayoshi Tokoro

Anton Tomsic

Aybala Tongut

Davide Tosi

Pasquale Totaro

Piergiorgio Tozzi

Nikolay Travin

Victor Tsang

Marko Turina

Akif Turna

James Tweddell

Naomichi Uchida

Yuichi Ueda

Murat Ugurlucan

Carin Van Doorn

Karel Van Praet

Dirk Van Raemdonck

Paul Van Schil

Panos Vardas

Gonzalo Varela

Praveen Varma

Dinakaran Vasudevan

Cecilia Vecoli

Prem Venugopal

Mathieu Vergnat

Vibeke Videm

Emmanuel Villa

Jan Vojacek

Luca Voltolini

Konstantin Von Aspern

Ulrich von Oppell

Kerem Vural

Andrew Waberski

Thorsten Walles

Isaac Wamala

Chunsheng Wang

Atsushi Watanabe

Taiju Watanabe

Stefan Watzka

Katrin Welcker

Stefan Welter

Luca Weltert

Olaf Wendler

Paul Werner

Douglas West

Ralph White

Randolph Wong

Ming-Ho Wu

Alain Wurtz

Qinzi Xu

Shun-Mao Yang

Can Yerebakan

Kanhua Yin

Francesco Zaraca

Daniel Zimpfer

Andrea Zuin

